# Medical students’ contribution to research; the scientific output of medical theses held in Moroccan medical schools during the last decade (2011-2021)

**DOI:** 10.1080/10872981.2023.2218677

**Published:** 2023-05-30

**Authors:** Youness Touissi, Ouajih Boulaich, Fatima Ezzahraa El Idrissi, Ghita Hjiej, Oussama Stitou, Hamza Belakbyer, Abderrazak Hajjioui, Maryam Fourtassi

**Affiliations:** a Faculty of Medicine and Pharmacy of Rabat, University Mohamed V, Rabat, Morocco; b Faculty of Medicine and Pharmacy of Tangier, Abdelmalek Essaâdi University, Tétouan, Morocco; c Faculty of Medicine and Pharmacy of Fez, Sidi Mohammed Ben Abdellah University, Fez, Morocco; d Laboratory of Life and Health Sciences, Faculty of Medicine and Pharmacy of Tangier, Abdelmalek Essaâdi University, Tétouan, Morocco; e Faculty of Medicine and Pharmacy of Marrakech, Cadi Ayyad University, Marrakech, Morocco

**Keywords:** Medical thesis, Research, medical student, Morocco, medical school

## Abstract

**Background:**

In order to obtain their medical degree, medical students in Morocco are required to carry out a research project and provide a thesis outlining its methodology and findings. However, little is known about the scientific output of these theses. The aim of the present study was to explore and evaluate the characteristics and publication patterns in scientific-indexed journals of medical theses written by medical students in Morocco.

**Methods:**

Data was extracted from registered theses between 2011 and 2021 in four medical schools that have an open-source documents archiving platform. Publication of these theses was assessed in 2022 using a search strategy in three indexed databases; Pubmed, Scopus and Web of science.

**Results:**

9807 theses were registered between 2011 and 2021, 41% of them in the faculty of medicine of Rabat. 99.1% of these theses were written in French, 61.7% were reporting a retrospective case series, and 38.9% of them covered surgical disciplines. 83 (0.8%) of the registered theses were published in a scientific-indexed journal, and half of the papers (49.4%) was written in French. The graduate student was the paper’s lead author in 54.2% of the papers. The articles driven from the theses were published after a mean delay of 1.49 ± 1.34 years and the targeted journals had a mean SJR score of 0.69 ± 1.21. The total number of detected citations of the 83 published papers was 216.

**Conclusion:**

The publication rate of Moroccan medical theses is very low when compared to other countries, which leads to question the real benefit of this time and resources consuming educational activity.

## Introduction

Modern medicine relies on evidence extracted from scientific research, in order to make the best possible decisions about how to treat patients, which represents the principle of evidence-based medicine (EBM) [1]. Hence, introducing undergraduate students to scientific research would be a good preparation for them to understand the need and importance of EBM in everyday practice. Participating in research projects improves undergraduate medical students’ critical thinking skills, and has been associated with increased postgraduate research output [2,3]. For medical students, participating in research is either optional as it is the case in the United States of America and the United Kingdom, or mandatory as in Germany and Sweden [4,5].

A medical doctor’s thesis, on the other hand, might be the only opportunity to get involved in scientific research before entering clinical practice, as it represents an integral aspect of the medical school curriculum. The publication of a thesis as a peer-reviewed paper is considered to be the best evidence for the accomplished research work quality [6]. Also, the number of the paper’s citations, and the quality of targeted journals are more often used as bibliometric indicators to quantify an institution’s scientific activity [7,8]. If some educational systems do require a thesis in order to obtain the medical academic degree (M.D), publication of this thesis as a scientific paper is not always required. Consequently, there is an everlasting controversy regarding the scientific usefulness of undergraduate medical theses and whether they achieve or not their educational goals.

Medical theses publishing rates vary by country and institution. The discrepancy could be explained by a number of factors, including research funding, academic culture, and publishing expectations. According to a previous study, 32.7% of New Zealand undergraduate medical theses resulted in scientific publications [9]. In the Netherlands, 27.7% of medical students had at least one publication as a result of their mandatory research projects [10], whereas in Turkey, the overall publication rate of health sciences and medical theses was 11.9% [11]. However, information regarding the rates of publishing of medical theses in different countries is limited.

In Morocco, after completing their studies, medical students often spend an additional year preparing their thesis, writing, editing, and eventually presenting it in order to graduate. This process consumes a significant amount of time and resources, including supervisors’ mentoring, and other faculty members reading and judging the work [12]. Such efforts can only be justified by the fact that the medical thesis is a crucial opportunity to expand knowledge in general medicine and to initiate the future doctor into scientific research [13].

Hundreds of medical theses are defended every year. However, their scientific impact in Moroccan medical schools is still undefined. Since 2015, a nationwide reform of medical studies, primarily focused on the undergraduate curriculum, has been conducted to improve efficiency and attain international standards. However, there are still many unresolved issues that require immediate attention, such as the ‘medical thesis’ [12]. The aim of the present study was to assess and compare the scientific output of medical students through their theses in Moroccan medical schools over the last decade. Were targeted the four public medical schools which made their thesis databases available online, namely the faculties of medicine and pharmacy of Rabat, Marrakech, Fez, and Oujda.

## Methods

### Study design

This is a multicentric bibliometric study that was conducted across medical schools in Morocco over a period of 11 years. At the time of the study, Morocco counted 8 public and 4 private medical schools, but only 5 institutions had an archive of medical theses; the remaining 7 were young institutions whose students hadn’t graduated yet. Among these 5 older institutions, one (the faculty of medicine and pharmacy of Casablanca) didn’t have an electronic database of theses. Hence, only four public medical schools could be considered. The study included all medical theses defended in the faculty of medicine and pharmacy of Rabat, Marrakech, Fez, and Oujda.

### Data collection

We conducted a thorough search of all accessible theses from 1 January 2011 to 31 December 2021 using the online databases of the four medical schools (Rabat, Marrakech, Fez, and Oujda). In the faculty of medicine and pharmacy of Oujda, which is a relatively young institution, theses started to be defended only in 2016. For each year, we collected the theses pdf versions from each faculty and noted the students’ names, the titles, and the advisors. Then, for each thesis, we manually specified the medical specialty, the language, and categorized the thesis methodology into six main types: case reports, retrospective case series, cross-sectional studies, prospective cohort studies (descriptive/interventional, randomized or not), reviews (narrative/systematic) and resources material production.

### Publication tracking

In order to identify the theses that had been published in a peer-reviewed indexed journal, the students’ last name and first letter of their first name were used as search queries in PubMed, Scopus, and Web of science databases, added to the affiliation city of each medical school, with no time limit. When a match between the title of a prescreened article and the title of the thesis was found, we looked for a match between the content of the abstracts to identify a thesis publication. If the comparison of the title and the abstract was not conclusive, we proceeded to a full text comparison. When the student’s name, advisor, title, and abstract matched between the thesis and the identified article, it was deemed as a published thesis.

### Published theses analysis

For each relevant publication, we recorded the journal name and its Scientific Ranking Journal score at the time of publication, the year of publication, the student’s rank in the authors’ list, the language, and the number of citations. The Quality of targeted journals was qualified by Scimago Journal & Country Rank (SJR) because it covers many journals targeted by medical students that are not indexed in Clarivate’s Web of Science and therefore have no impact factor. The highest number of citations from the Web of science, Scopus, and Pubmed databases was used to indicate the number of citations for each article.

The present study was examined and approved by the ethics committee of the faculty of medicine and pharmacy of Tangier; Morocco.

## Results

### General description of the analyzed theses

Over 11 years (2011–2021), a total of 9807 were produced by graduating students in four faculties of medicine. 99.1% of these theses were written and presented in French. The most used research methodology was ‘retrospective case series’ representing 61.7% of all analyzed theses, followed by ‘reviews’ (12.5%) and ‘case reports’ (11.5%). As for the specialty domain, surgical specialties top ranked covering 38.9% of all analyzed theses, followed by mother and child health (22%), then medical specialties (18.6%). Medical education as a subject of the thesis work came last covering only 1.3% of all defended theses. Theses’ characteristics for each institution are summarized in Table 1.

**Table 1.** General characteristics of medical theses in the four explored faculties of medicine and pharmacy (2011–2021).

Items	Faculty of medicine & pharmacy of Rabat N (%)	Faculty of medicine & pharmacy of Fez N (%)	Faculty of medicine & pharmacy of Marrakech N (%)	Faculty of medicine & pharmacy of Oujda N (%)	Total N (%)
**Language**					
French	4002 (99.3)	2465 (98.9)	2078 (99.0)	1179 (99.0)	9724 (99.1)
English	20 (.5)	18 (.8)	18 (.9)	10 (.9)	66 (0.7)
Arabic	6 (.2)	8 (.3)	2 (.1)	1 (.1)	17 (0.2)
**Methodology**					
Retrospective case series	2189 (54.3)	1589 (63.8)	1609 (76.7)	668 (56.1)	6055 (61.7)
Case reports	497 (12.3)	355 (14.2)	46 (2.2)	227 (19.1)	1125 (11.5)
Prospective cohort study	274 (6.9)	188 (7.6)	156 (7.4)	18 (1.5)	636 (6.5)
Cross-sectional study	96 (2.4)	203 (8.1)	228 (1.9)	54 (4.5)	581 (6.0)
Reviews	960 (23.8)	52 (2.1)	6 (.3)	209 (17.6)	1227 (12.5)
Resource material creation	12 (.3)	104 (4.2)	53 (2.5)	14 (1.2)	183 (1.8)
**Specialty domains**					
Surgical specialties	1261 (31.3)	1041 (41.8)	914 (43.6)	594 (49.9)	3810 (38.9)
Medical specialties	612 (15.3)	562 (22.7)	446 (21.3)	205 (17.2)	1825 (18.6)
Mother and child health	1182 (29.3)	440 (17.7)	281 (13.4)	250 (21.1)	2153 (22.0)
Fundamental sciences	636 (15.8)	196 (7.8)	237 (11.3)	59 (4.9)	1128 (11.5)
Emergency-Intensive care	296 (7.3)	198 (7.9)	155 (7.4)	69 (5.7)	718 (7.3)
Medical education	20 (.5)	44 (1.7)	55 (2.5)	15 (1.2)	134 (1.3)
Miscellaneous	21 (.5)	10 (.4)	10 (.5)	-	41 (0.4)
**Total**	4028 (100)	2491 (100)	2098 (100)	1190 (100)	9807(100)

### Characteristics of published theses

Only 83 theses generated a published article out of the 9805 theses that were collected over a decade, with a publishing rate of 0.8% across the four investigated institutions. Only two theses generated more than one published article. The faculty of medicine and pharmacy of Marrakech had the greatest publishing rate with 1.4%, followed by the faculty of Fez (1.0%), the faculty of Rabat (0.6%), and finally the faculty of Oujda (0.3%).

**Figure 1.**
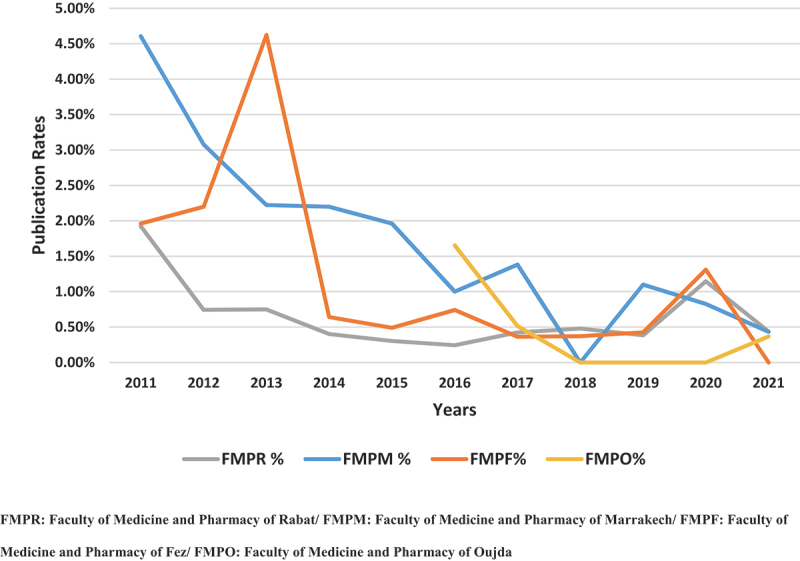
Evolution of the publication rate through the years in the four medical faculties.

The publication rate of each university for each year is displayed in Figure 1. 2011 was the most successful year, with 14 articles published, while 2018 was the least productive, with just three articles published across all institutions. There were 41 articles published in French and 42 in English, with 37 of the latter coming from French theses that had to be translated before publishing while the remaining 5 articles were previously written in English.

Oncology was the most represented discipline in the published theses, with seven articles (8.4%), followed by urology and psychiatry with six articles each (7.2%), then ophthalmology with five (6%). Also, 71 students (86.6%) shared the same affiliation of their supervisors on the published paper.

**Figure 2.**
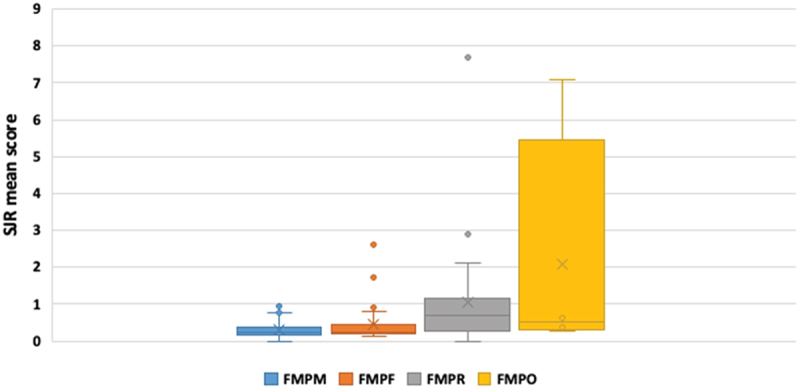
Comparison of published theses among the four medical faculties regarding the SJR score means.

The SJR score of the targeted journals ranged from 0.1 to 7.69, and its means were higher in papers published from the Faculties of Oujda and Rabat (Cf. Figure 2). The ‘Pan African Medical Journal’ was the most targeted journal, publishing 17 of the 83 papers (20.5%). The average number of citations per article was 2.6, the greatest number of citations an article received among all published papers was 24. And 35 published articles (42%) were never referenced.

FMPR: Faculty of Medicine and Pharmacy of Rabat/FMPM: Faculty of Medicine and Pharmacy of Marrakech/FMPF: Faculty of Medicine and Pharmacy of Fez/FMPO: Faculty of Medicine and Pharmacy of Oujda.

Despite two papers that were published before the theses defense, the average time to publish was a year and a half after graduation, with a maximum of 6 years. As shown in Table 2, the student author of the thesis was occupying the lead author position in the published article in only 54.2% of the cases.

**Table 2.** General characteristics of medical theses in the four explored faculties of medicine and pharmacy (2011–2021).

Items	Faculty of medicine & pharmacy of Rabat N (%)	Faculty of medicine & pharmacy of Fez N (%)	Faculty of medicine & pharmacy of Marrakech N (%)	Faculty of medicine & pharmacy of Oujda N (%)	Total
**Published papers**	24 (.6)	25 (1.0)	30 (1.4)	4 (.3)	83 (0.8)
**Student’s gender**					
Men	8 (33.3)	13 (52.0)	14 (46.7)	2 (5.0)	37 (44.6)
Women	16 (66.7)	12 (48.0)	16 (53.3)	2 (5.0)	46 (55.4)
**Author rank of the student**					
First author	14 (58.4)	13 (52.0)	16 (53.3)	2 (5.0)	45 (54.2)
Second author	7 (29.1)	7 (28.0)	3 (1.0)	2 (5.0)	19 (22.9)
Third author or further	3 (12.5)	5 (2.0)	11 (36.7)	0 (.90)	19 (22.9)
**Language of publication**					
French	5 (2.8)	17 (68.0)	19 (63.3)	0 (.0)	41 (49.4)
English	19 (79.2)	8 (32.0)	11 (36.7)	4 (1.0)	42 (50.6)
**Journal SJR (Means ± SD)**	1.15 ± 1.60	45 ± .56	33 ± .24	2.09 ± 3.32	0.69 ± 1.21
**Delay of publication in years (Means ± SD)**	1.08 ± 1.8	2.28 ± 1.21	2.10 ± 1.64	51 ± 1.0	1.49 ± 1.34
**Number of Citations**	105	38	60	13	216

SJR: Scientific Journal Ranking; SD: Standard Deviation

## Discussion

The present bibliometric study revealed interesting and challenging results regarding the scientific outcome of medical theses defended in Moroccan medical schools. Indeed, less than 1% of those theses find their way to publication in scientific journals, which is far lower than in other countries such as France (17%), New Zealand (32.7%), Netherlands (27.7%), Finland (23.8%), Turkey (11.9%), or even in Tunisia (13.4%) which has a similar educational system [6,9–11,14,15]. Not only the publication rate is low, but it continues to fall year after year, despite an increasing number of students’ enrolments and faculty members recruitment in each institution, reflecting the fact that scientific publication might not be a top priority in the Moroccan medical faculties.

It seems that pushing students to publish their theses is a complex issue. Due to the limited number of published theses, and the scarcity of data related to graduating students, it was not possible to statically explore the factors that lead to publishing. Nonetheless, there are a variety of plausible explanations for this occurrence. Historically, medical students have viewed the completion of an M.D. thesis as an obligation or a necessary activity to achieve their primary goal, that is graduation. As a result, the majority of students do not attempt to investigate difficult or novel topics, but rather recycle previous theses with minor modifications [16], leading to high levels of plagiarism [17]. Additionally, students who do not work in the same department as their thesis supervisor after they graduate are less likely to publish the results of their findings because students don’t get enough training in how to write articles while they are in medical school, and it’s unlikely that a supervisor will help them after graduation [18]. In the present study, 86.6% of graduates who published their medical theses, were linked to the same department as their advisor, allowing them to publish their work even after a 6-year delay. Another issue to note is that students get no formal research training or seminars on how to produce a paper throughout their years of studies, and they work on their thesis solely on their own initiative and with the help of their peers [12,19]. Therefore, a poor publishing culture prevails among medical students.

Language is also a major problem; according to our findings, only 5 theses were written in English and resulted in published articles in English. The rest of the 78 articles were based on theses written and defended in French, which is the main teaching language in medical education in Morocco [9]. Considering that English is now recognized as the primary language of research and scientific publishing, it must be mastered in order to increase one’s chances to have a paper published in indexed high quality scientific journals [5].

A prior study in one of our included institutions (the faculty of medicine of Fez) pointed out significant flaws in the methodological rigor of medical theses [13]. These methodological inadequacies also explain the low publishing rate of medical theses in Morocco. Indeed, even the theses that did get published in our study were aimed at journals with low SJR scores, and almost half of them were never cited.

In contrast to numerous studies revealing that women are underrepresented in academic work, especially medical research [20,21], female students were more represented in published theses than males (55.4%). This could be fairly explained by the higher number of female medical students representing around two-thirds of medical students in Morocco [22].

Regardless of the reasons provided, the thesis position in the qualifying process of medical studies should be clearly determined. If a research experience is required, medical schools must act quickly to ensure the quality and authenticity of the experience by peer review publishing, which is an objective assessment of whether or not the objectives of the thesis have been met.

Many measures could promote research and the scientific spirit among medical students, as many studies have shown their rising interest in scientific research and their readiness to engage in research activities [23,24]. Creating joined MD-PhD programs for students desiring to pursue a research career [12]; and determining ‘publishing the thesis’ as a primary requirement to enter such a path would encourage students to publish more. Also, assigning young students research projects and teaching them the basics of bibliographical search, scientific writing and editing would help push them to get their work published. And of course, promoting the use of English in written communication and raising awareness about plagiarism would also play a key role in enhancing students’ publishing potential [25]. Finally, such goals cannot be achieved without appropriate supervision, with close guidance and substantive support, which is time and effort consuming [26,27].

Publishing the outcomes of theses research in peer-reviewed journals is probably the most objective approach to establish if the whole procedure is effective and beneficial [5]. However, research productivity of medical students cannot be measured solely by the number of published theses. Indeed, students may engage in research projects as part of extracurricular activities; and disseminate their results in the form of posters at national conferences; or serve a local purpose. In some cases, we found published theses that did not include the student’s name, which might indicate a low contribution to the work. Even if this does not explain the stated publication levels, it raises questions about the utility of such assignments as writing a thesis, and the manner in which they are implemented. Additional research should investigate the perspectives of students and supervisors regarding the importance of theses in the course of medical studies and their motivation for going through the scientific publication process.

## Limitations

The present work has some limitations to take into consideration when drawing conclusions. The bibliometric search was limited to the three most common databases; Scopus, Web of Science, and PubMed. Hence, some published articles indexed elsewhere could have been missed. It is worth noting that even recent theses (up to 2021) were included, and some of these might be in the process of scientific publication, leading to underestimation of the overall publication rate. Despite these limitations, our findings may be valuable when considering potential educational and training initiatives in Moroccan medical schools and other educational systems that may mandate a thesis as part of the curriculum.
